# Trajectory‐Based Clustering to Identify Asthma Subgroups Responsive to the Selective CXCR2 Antagonist, AZD5069

**DOI:** 10.1111/all.70140

**Published:** 2025-11-02

**Authors:** Khezia Asamoah, Freda Yang, Ian M. Adcock, Dragana Vuckovic, Mohib Uddin, Kian Fan Chung, Marc Chadeau‐Hyam

**Affiliations:** ^1^ MRC Centre for Environment and Health & Department of Epidemiology and Biostatistics Faculty of Medicine, School of Public Health, Imperial College London London UK; ^2^ National Heart and Lung Institute Imperial College London London UK; ^3^ Data Science Institute, Department of Computing Imperial College London London UK; ^4^ AstraZeneca BioPharmaceuticals R&D Gothenburg Sweden; ^5^ Royal Brompton and Harefield Hospital London UK

**Keywords:** asthma, asthma treatment, biomarkers

To the Editor,

Neutrophilic asthma is a subphenotype of asthma that affects 20%–30% of all adult asthma patients and is typically harder to treat [1]. Usually driven by type 1 inflammation and external triggers, it is associated with a complex clinicopathobiology [2, 3]. AZD5069, a selective CXCR2 antagonist developed to block neutrophil trafficking without compromising immunity [4, 5], showed biological effects by lowering neutrophil counts in blood, sputum and bronchial biopsies in severe asthma [6]. However, it failed to improve clinical outcomes [7]. In this study, we reanalysed the data using a trajectory‐based clustering approach to identify potential responder subgroups to AZD5069 among patients with presumed neutrophilic asthma.

The data were obtained from a multicentre, double‐blind, placebo‐controlled, dose‐ranging Phase 2b trial involving 640 patients randomised to four arms: AZD5069 at 5, 15 or 45 mg, or matched placebo. The study was done in accordance with the principles of the Declaration of Helsinki, the International Conference on Harmonisation guidelines for good clinical practice, and applicable regulatory requirements. All patients provided written informed consent before any study‐specific assessments were done. 422 participants had complete longitudinal measurements for key variables across required timepoints and their characteristics are reported in Table S1. A broad panel of 22 blood biomarkers was collected. To identify distinct patterns in patient response trajectories, a consensus clustering framework (using dynamic time warping) was used on timepoints from 1 to 6 months. Clustering was based on raw values for: pre‐bronchodilator FEV_1_ (L), Asthma Control Questionnaire (ACQ)‐5 and blood neutrophil count (NEUT).

Three distinct trajectory clusters were identified based on changes in asthma‐related outcomes and inflammatory markers (Figure 1). Cluster 3 was characterised by a higher proportion of female patients, a greater prevalence of high baseline BMI, a higher baseline severe exacerbation rate and a lower FEV1/FVC ratio, together with elevated blood neutrophil counts compared with the other clusters. Each cluster included participants from both placebo and treatment arms. The treatment arm across all three clusters saw a significant reduction in NEUT or neutrophil count as a percentage of leukocytes (NEUTLE) (Figure 1, Table S2). Cluster 2 (green) also showed a significant reduction in ACQ‐5 (*p* = 0.019), with 42% showing a clinically important reduction of ≥ 0.5 in ACQ score (Table S3). In contrast, Cluster 3 (blue) remained relatively refractory regarding FEV_1_ change. Trajectory groups differed significantly in several baseline characteristics (Table S4).

**FIGURE 1:**
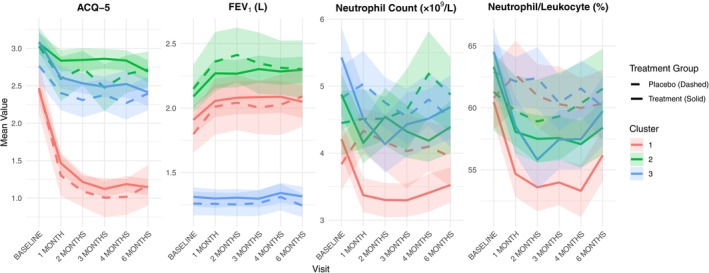
Longitudinal trends of clinical biomarkers across treatment and placebo groups stratified by cluster. Mean values with 95% confidence intervals are shown for four metrics (ACQ‐5, FEV1, NEUT, NEUTLE) over time from baseline to 6 months. Solid lines represent the treatment group, while dashed lines represent the placebo group. Clusters (1–3) are colour‐coded, highlighting differential responses among patient subgroups.

Figure 2 displays log‐fold changes in biomarker levels across placebo and treatment arms for each trajectory cluster; however there was no significant difference between treatment arms per cluster post‐correction (Table S5). In Cluster 3, both placebo and treatment arms showed a reduction in monocyte levels (MONOLE), while in Clusters 1 and 2, treatment arms showed increases in eosinophils (EOS, EOSLE). Changes in neutrophil‐to‐lymphocyte ratio (NLR) from baseline to six months varied across clusters (Figure S1 and Table S6). In Clusters 1 and 2, treatment arms saw a decrease in NLR (*p* = < 0.001 and < 0.003, respectively). By contrast, the Cluster 3 treatment arm showed no reduction in NLR, which may reflect the influence of clinical features such as female predominance and obesity that contribute to greater asthma severity.

**FIGURE 2:**
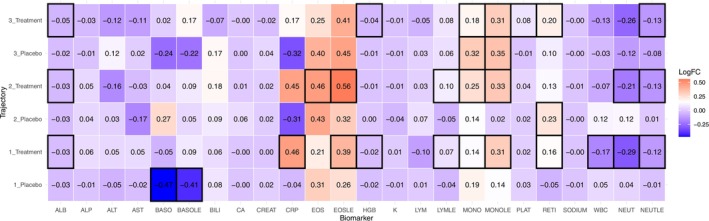
Heatmap of log‐fold changes in biomarker levels by treatment group and trajectory cluster. Each cell represents the log‐fold change value for a specific biomarker (*x*‐axis) within a treatment or placebo group stratified by trajectory cluster (*y*‐axis). Colour intensity indicates the direction and magnitude of change (red = increased, blue = decreased), and black borders highlight statistically significant values between baseline and 6‐month mark ‐ Bonferroni correction.

The findings of 3 discrete patterns of response highlight the heterogeneity within ‘neutrophilic asthma’, helping to explain the neutral overall results of the parent trial [7]. Nonetheless, treatment produced clinically significant improvements in ACQ‐5 with higher proportions of participants on active treatment achieving a minimal clinically important difference of ≥ 0.5 in ACQ for clusters 2 and 3. The emergence of a treatment‐responsive subgroup despite a negative headline outcome underscores the value of trajectory analysis in respiratory drug development. Identifying a Cluster 2‐like phenotype prospectively could enrich future trials for likely responders, making them precise, faster and more informative; however, with such specificity, those who do not fit the cluster profile but could nevertheless potentially benefit from the treatment may be overlooked. The divergent biomarker shifts, such as eosinophil rises confined to active‐treatment arms in Clusters 1 and 2, suggest that CXCR2 blockade may modulate or potentiate other immune pathways beyond neutrophil trafficking. Mechanistically, CXCR2 antagonism has been shown to induce a compensatory upregulation of neutrophil‐modulatory cytokines, in both non‐human primates [5] and severe asthma patients [6]. The interactive nature of these immune pathways following CXCR2 antagonism tentatively suggests a feedback mechanism in which cytokine‐mediated immune skewing may promote Type 2 (T2) signalling, thereby increasing the likelihood of an eosinophilic predominant T2‐high inflammation observed.

This study has some limitations: a restricted biomarker panel overlooking key mechanistic signals; exclusion of eosinophilic phenotypes and heavy smokers limiting generalisability. In the future, the clustering framework can be applied in other clinical trials and related studies, while broader omics profiling and airway sampling would help explain why clusters differ in treatment response to emerging therapeutics.

## Author Contributions

K.A. conducted the analysis and drafted the initial manuscript. M.U. and M.C.‐H. contributed to writing and critical revision of the letter with M.U. providing the data. F.Y., I.M.A., D.V. and K.F.C. reviewed the manuscript and provided feedback. All authors approved the final version.

## Conflicts of Interest

Dr. Chung has received honoraria for participating in Advisory Board meetings of GSK, AZ, Novartis, Roche, Merck, Trevi, Nocion, Shionogi and Rickett‐Beckinson and has also been renumerated for speaking engagements for GSK, Novartis and AZ. He holds research grants from GSK and Merck, paid to his institution. F.Y. has received honoraria for attending meetings and speaker fees from AZ and GSK, and is a member of the British Thoracic Society Asthma Advisory Group. M.U. is an employee and holds shares in AstraZeneca. M.C.‐H. holds shares in the O‐SMOSE company and has no conflicts of interest to disclose. Consulting activities conducted by the company are independent of the present work. The other authors declare no conflicts of interest.

## Supporting information


**Appendix S1:** all70140‐sup‐0001‐Supinfo.docx.
**Table S1:** Baseline demographic and clinical characteristics by treatment group.
**Table S2:** Table showing mean (and standard deviation) change in cluster variables at baseline and 6 months. *p*‐values signal whether the change at baseline and 6 months is significant between treatment and placebo arms.
**Table S3:** Percentage of patients per cluster and treatment arm achieving ≥ 0.5 drop in ACQ‐5.
**Table S4:** Baseline characteristics stratified by trajectory clusters (Cluster 1, 2 and 3).
**Table S5:** *P*‐values from unpaired *t*‐tests (with Bonferroni correction) comparing log fold change between placebo and treatment groups within each trajectory cluster, for each biomarker.
**Table S6:** *P*‐values from paired tests comparing neutrophil‐to‐lymphocyte ratio (NLR) at baseline and six months within each cluster and treatment arm.
**Figure S1:** Mean Neutrophil‐to‐Lymphocyte Ratio (NLR) at Baseline and 6 Months by Trajectory Cluster and Treatment Group. Line plot displays changes in mean NLR over time across trajectory clusters (1–3), stratified by treatment (solid lines) and placebo (dashed lines) groups.

## Data Availability

The data that support the findings of this study are available on request from the corresponding author. The data are not publicly available due to privacy or ethical restrictions.
