# Supplementary material for: Trajectory‐Based Clustering to Identify Asthma Subgroups Responsive to the Selective CXCR2 Antagonist, AZD5069
Source: Allergy. 2025 Nov 2;81(1):297–9. doi: 10.1111/all.70140 (PMC12773661; doi:10.1111/all.70140)
Supplement: Supplementary file 1 — Appendix S1: all70140‐sup‐0001‐Supinfo.docx. Table S1: Baseline demographic and clinical characteristics by treatment group. Table S2: Table showing mean (and standard deviation) change in cluster variables at baseline and 6 months. p‐values signal whether the change at baseline and 6 months is significant between treatment and placebo arms. Table S3: Percentage of patients per cluster and treatment arm achieving ≥ 0.5 drop in ACQ‐5. Table S4: Baseline characteristics stratified by trajectory clusters (Cluster 1, 2 and 3). Table S5: P‐values from unpaired t‐tests (with Bonferroni correction) comparing log fold change between placebo and treatment groups within each trajectory cluster, for each biomarker. Table S6: P‐values from paired tests comparing neutrophil‐to‐lymphocyte ratio (NLR) at baseline and six months within each cluster and treatment arm. Figure S1: Mean Neutrophil‐to‐Lymphocyte Ratio (NLR) at Baseline and 6 Months by Trajectory Cluster and Treatment Group. Line plot displays changes in mean NLR over time across trajectory clusters (1–3), stratified by treatment (solid lines) and placebo (dashed lines) groups. [file ALL-81-297-s001.docx]

**Supplementary file:**

**Trajectory based clustering to identify asthma subgroups following treatment with the selective CXCR2 antagonist, AZD5069**

Khezia Asamoah^1^, Freda Yang^3^, Ian M Adcock^2,3^, Dragana Vuckovic^1^, Mohib Uddin^5^, Kian Fan Chung^2,3,4^, Marc Chadeau-Hyam^1†^

**Data and methodology additional information**

Key inclusion criteria included a physician-diagnosed asthma for ≥12 months, uncontrolled symptoms despite medium- to high-dose ICS plus LABA, ≥1 severe exacerbation in the past year, and a pre-bronchodilator FEV₁ of 30–85% predicted. Objective evidence of variable airflow obstruction and stable background therapy for ≥30 days were also required. Key exclusion criteria included biomarkers indicative of eosinophilic or allergic asthma (blood eosinophils > 0.5 × 10⁹/L or serum IgE > 750 kIU/L), use of other biologics or 5-lipoxygenase inhibitors, active infections, baseline absolute neutrophil count < 2.7 × 10⁹/L at enrolment (Visit 1), and a smoking history of more than 10 pack-years.

Dynamic Time Warping (DTW) was used to align and compare trajectories, allowing flexibility in the timing and shape of individual responses. Clustering was done using a resampling-based approach: 100 subsamples were generated (each 50% of the data), and clustering was run within each. For each value of k (number of clusters), a stability matrix was calculated by recording how often individuals were grouped together across subsamples, normalised by how often they were co-included and a consensus score was calculated. The number of clusters that maximised the consensus score was selected with Partitioning Around Medoids (PAM) then applied to the stability matrix.

**Supplementary Table 1: Baseline demographic and clinical characteristics by treatment group.**

|  |  | **Overall** | **AZD15** | **AZD45** | **AZD5** | **PLACEBO** |
| --- | --- | --- | --- | --- | --- | --- |
| **n** |  | 422 | 104 | 103 | 102 | 113 |
| **Gender (%)** | Female | 298 (70.6) | 73 (70.2) | 71 (68.9) | 70 (68.6) | 84 (74.3) |
|  | Male | 124 (29.4) | 31 (29.8) | 32 (31.1) | 32 (31.4) | 29 (25.7) |
| **Baseline BMI (%)** | 0 < x ≤ 19 | 5 (1.2) | 2 (1.9) | 1 (1.0) | 2 (2.0) | 0 (0.0) |
|  | 19 < x ≤ 25 | 117 (27.7) | 30 (28.8) | 23 (22.3) | 28 (27.5) | 36 (31.9) |
|  | 25 < x ≤ 30 | 173 (41.0) | 38 (36.5) | 41 (39.8) | 45 (44.1) | 49 (43.4) |
|  | 30 < x ≤ 80 | 127 (30.1) | 34 (32.7) | 38 (36.9) | 27 (26.5) | 28 (24.8) |
| **Age (%)** | 15 < x ≤ 25 | 7 (1.7) | 5 (4.8) | 0 (0.0) | 2 (2.0) | 0 (0.0) |
|  | 25 < x ≤ 35 | 27 (6.4) | 9 (8.7) | 9 (8.7) | 3 (2.9) | 6 (5.3) |
|  | 35 < x ≤ 45 | 79 (18.7) | 18 (17.3) | 20 (19.4) | 18 (17.6) | 23 (20.4) |
|  | 45 < x ≤ 55 | 115 (27.3) | 23 (22.1) | 35 (34.0) | 25 (24.5) | 32 (28.3) |
|  | 55 < x ≤ 65 | 142 (33.6) | 39 (37.5) | 30 (29.1) | 42 (41.2) | 31 (27.4) |
|  | 65 < x ≤ 75 | 46 (10.9) | 8 (7.7) | 8 (7.8) | 11 (10.8) | 19 (16.8) |
|  | 75+ | 6 (1.4) | 2 (1.9) | 1 (1.0) | 1 (1.0) | 2 (1.8) |
| **Smoking Status (%)** | Former | 113 (26.8) | 19 (18.3) | 30 (29.1) | 31 (30.4) | 33 (29.2) |
|  | Never | 309 (73.2) | 85 (81.7) | 73 (70.9) | 71 (69.6) | 80 (70.8) |
| **Baseline OCS Use (%)** | No | 400 (94.8) | 96 (92.3) | 98 (95.1) | 98 (96.1) | 108 (95.6) |
|  | Yes | 22 (5.2) | 8 (7.7) | 5 (4.9) | 4 (3.9) | 5 (4.4) |
| **Severe Exacerbation Rate (mean (SD))** | | 0.18 (0.47) | 0.24 (0.53) | 0.17 (0.49) | 0.13 (0.39) | 0.18 (0.45) |
| **Pack Years (mean (SD))** |  | 6.39 (3.89) | 7.16 (4.05) | 5.53 (3.45) | 6.52 (4.33) | 6.61 (3.77) |
| **Animal Allergy (%)** | No | 373 (88.4) | 89 (85.6) | 94 (91.3) | 90 (88.2) | 100 (88.5) |
|  | Yes | 49 (11.6) | 15 (14.4) | 9 (8.7) | 12 (11.8) | 13 (11.5) |
| **House Dust Mite Allergy (%)** | No | 323 (76.5) | 72 (69.2) | 84 (81.6) | 83 (81.4) | 84 (74.3) |
|  | Yes | 99 (23.5) | 32 (30.8) | 19 (18.4) | 19 (18.6) | 29 (25.7) |
| **Mould Allergy (%)** | No | 391 (92.7) | 92 (88.5) | 96 (93.2) | 96 (94.1) | 107 (94.7) |
|  | Yes | 31 (7.3) | 12 (11.5) | 7 (6.8) | 6 (5.9) | 6 (5.3) |
| **Other Allergy (%)** | No | 361 (85.5) | 89 (85.6) | 89 (86.4) | 88 (86.3) | 95 (84.1) |
|  | Yes | 61 (14.5) | 15 (14.4) | 14 (13.6) | 14 (13.7) | 18 (15.9) |
| **Seasonal Allergy (%)** | No | 335 (79.4) | 78 (75.0) | 82 (79.6) | 83 (81.4) | 92 (81.4) |
|  | Yes | 87 (20.6) | 26 (25.0) | 21 (20.4) | 19 (18.6) | 21 (18.6) |
| **FEV1/FVC (mean (SD))** |  | 0.63 (0.13) | 0.65 (0.14) | 0.63 (0.12) | 0.62 (0.12) | 0.63 (0.13) |
| **Blood Eosinophil Count(mean (SD))** | | 0.19 (0.17) | 0.20 (0.19) | 0.19 (0.14) | 0.19 (0.18) | 0.19 (0.16) |
| **Blood Neutrophil Count (mean (SD))** | | 4.67 (1.73) | 4.71 (1.79) | 4.77 (1.70) | 4.84 (1.89) | 4.41 (1.52) |

*Former smokers were defined as individuals with any history of cigarette use, quantified in pack-years.*

**Supplementary Table 2: Table showing mean (and standard deviation) change in cluster variables at baseline and 6 months. P-values signal whether the change at baseline and 6 months is significant between treatment and placebo arms.**

| **Trajectory** | **Arm** | **ACQ-5: Baseline** | **ACQ-5: 6 months** | **ACQ-5: p-values** | **FEV1: Baseline** | **FEV1: 6 months** | **FEV1:**  **p-values** | **NEUT:**  **Baseline** | **NEUT:**  **6 months** | **NEUT**  **p-values** | **NEUTLE:Baseline** | **NEUTLE:6 months** | **NEUTLE:**  **p-value** |
| --- | --- | --- | --- | --- | --- | --- | --- | --- | --- | --- | --- | --- | --- |
| **1** | **Placebo** | **2.47 (1.05)** | **1.17 (0.816)** | **0.943** | **1.8 (0.434)** | **2.09**  **(0.704)** | **0.121** | **3.84 (1.18)** | **3.94 (1.34)** | **<0.001** | **60.8 (7.77)** | **60.3 (8.30)** | **0.002** |
|  | **Treatment** | **2.44 (0.886)** | **1.15 (0.613)** |  | **1.91 (0.626)** | **2.05 (0.707)** |  | **4.21 (1.45)** | **3.52 (1.48)** |  | **60.5 (9.35)** | **56.2 (11.6)** |  |
| **2** | **Placebo** | **3.03 (0.684)** | **2.72 (0.664)** | **0.019** | **2.15 (0.505)** | **2.30 (0.612)** | **0.052** | **4.45 (1.31)** | **4.87 (1.52)** | **0.074** | **61.3 (8.67)** | **61.5 (8.84)** | **<0.001** |
|  | **Treatment** | **3.07 (0.710)** | **2.69 (0.760)** |  | **2.09 (0.476)** | **2.30 (0.525)** |  | **4.88 (1.64)** | **4.39 (2.01)** |  | **63.3 (8.42)** | **58.4 (11.3)** |  |
| **3** | **Placebo** | **2.76 (0.805)** | **2.40 (0.814)** | **0.009** | **1.26 (0.333)** | **1.24 (0.326)** | **0.412** | **4.82 (1.73)** | **4.52 1.57)** | **0.077** | **62.9 (8.38)** | **60.1 (9.67)** | **0.042** |
|  | **Treatment** | **3.09 (0.709)** | **2.41 (0.880)** |  | **1.31 (0.354)** | **1.31 (0.356)** |  | **5.43 (2.11)** | **4.69 (2.25)** |  | **64.5 (9.31)** | **59.7 (13.2)** |  |

**Supplementary Table 3: Percentage of patients per cluster and treatment arm achieving ≥0.5 drop in ACQ-5.**

| **Cluster** | **Treatment Arm** | **Percentage** | **p-value** |
| --- | --- | --- | --- |
| 1 | Placebo | 68.6 | 0.259 |
| 1 | Treatment | 79.5 |  |
| 2 | Placebo | 34.5 | 0.597 |
| 2 | Treatment | 42.2 |  |
| 3 | Placebo | 39.2 | 0.078 |
| 3 | Treatment | 56.3 |  |

**Supplementary Table 4: Baseline characteristics stratified by trajectory clusters (Cluster 1, 2, and 3).**

|  |  | **Cluster 1** | **Cluster 2** | **Cluster 3** | **p** |
| --- | --- | --- | --- | --- | --- |
| **n** |  | 157 | 130 | 135 |  |
| **Gender (%)** | Female | 98 (62.4) | 82 (63.1) | 118 (87.4) | <0.001 |
|  | Male | 59 (37.6) | 48 (36.9) | 17 (12.6) |  |
| **Treatment Group (%)** | AZD15 | 40 (25.5) | 36 (27.7) | 28 (20.7) | 0.112 |
|  | AZD45 | 44 (28.0) | 30 (23.1) | 29 (21.5) |  |
|  | AZD5 | 38 (24.2) | 35 (26.9) | 29 (21.5) |  |
|  | PLACEBO | 35 (22.3) | 29 (22.3) | 49 (36.3) |  |
| **Baseline BMI (%)** | 0 < x ≤ 19 | 3 (1.9) | 1 (0.8) | 1 (0.7) | 0.013 |
|  | 19 < x ≤ 25 | 48 (30.6) | 37 (28.5) | 32 (23.7) |  |
|  | 25 < x ≤ 30 | 65 (41.4) | 63 (48.5) | 45 (33.3) |  |
|  | 30 < x ≤ 80 | 41 (26.1) | 29 (22.3) | 57 (42.2) |  |
| **Age (%)** | 15 < x ≤ 25 | 2 (1.3) | 4 (3.1) | 1 (0.7) | <0.001 |
|  | 25 < x ≤ 35 | 13 (8.3) | 12 (9.2) | 2 (1.5) |  |
|  | 35 < x ≤ 45 | 31 (19.7) | 35 (26.9) | 13 (9.6) |  |
|  | 45 < x ≤ 55 | 39 (24.8) | 40 (30.8) | 36 (26.7) |  |
|  | 55 < x ≤ 65 | 46 (29.3) | 35 (26.9) | 61 (45.2) |  |
|  | 65 < x ≤ 75 | 20 (12.7) | 4 (3.1) | 22 (16.3) |  |
|  | 75+ | 6 (3.8) | 0 (0.0) | 0 (0.0) |  |
| **Smoking Status (%)** | Former | 41 (26.1) | 35 (26.9) | 37 (27.4) | 0.969 |
|  | Never | 116 (73.9) | 95 (73.1) | 98 (72.6) |  |
| **Baseline OCS Use (%)** | No | 146 (93.0) | 125 (96.2) | 129 (95.6) | 0.433 |
|  | Yes | 11 (7.0) | 5 (3.8) | 6 (4.4) |  |
| **Crude Baseline Severe Exacerbation Rate (mean (SD))** | | 0.11 (0.36) | 0.18 (0.48) | 0.26 (0.57) | 0.043 |
| **Pack Years (mean (SD))** |  | 7.80 (4.69) | 5.51 (3.29) | 5.65 (2.95) | 0.013 |
| **Animal Allergy (%)** | No | 135 (86.0) | 117 (90.0) | 121 (89.6) | 0.493 |
|  | Yes | 22 (14.0) | 13 (10.0) | 14 (10.4) |  |
| **House Dust Mite Allergy (%)** | No | 124 (79.0) | 103 (79.2) | 96 (71.1) | 0.196 |
|  | Yes | 33 (21.0) | 27 (20.8) | 39 (28.9) |  |
| **Mould Allergy (%)** | No | 145 (92.4) | 124 (95.4) | 122 (90.4) | 0.29 |
|  | Yes | 12 (7.6) | 6 (4.6) | 13 (9.6) |  |
| **Other Allergy (%)** | No | 131 (83.4) | 119 (91.5) | 111 (82.2) | 0.063 |
|  | Yes | 26 (16.6) | 11 (8.5) | 24 (17.8) |  |
| **Seasonal Allergy (%)** | No | 120 (76.4) | 105 (80.8) | 110 (81.5) | 0.509 |
|  | Yes | 37 (23.6) | 25 (19.2) | 25 (18.5) |  |
| **FEV1/FVC (mean (SD))** |  | 0.63 (0.13) | 0.69 (0.10) | 0.57 (0.12) | <0.001 |
| **Blood Eosinophil Count (mean (SD))** | | 0.21 (0.18) | 0.17 (0.16) | 0.19 (0.16) | 0.146 |
| **Blood Neutrophil Count (mean (SD))** | | 4.13 (1.40) | 4.78 (1.59) | 5.21 (2.00) | <0.001 |

**Supplementary Table 5: P-values from unpaired t-tests (with Bonferroni correction) comparing log fold change between placebo and treatment groups within each trajectory cluster, for each biomarker.**

|  | 1 | 2 | 3 |
| --- | --- | --- | --- |
| ALB | 0.9674 | 0.9234 | 0.4771 |
| ALP | 0.4494 | 0.9869 | 0.8025 |
| ALT | 0.5660 | 0.5535 | 0.0894 |
| AST | 0.6208 | 0.5535 | 0.2444 |
| BASO | 0.1454 | 0.6208 | 0.3922 |
| BASOLE | 0.1430 | 0.9671 | 0.2065 |
| BILI | 0.9671 | 0.7872 | 0.2270 |
| CA | 0.9674 | 0.6208 | 0.9728 |
| CREAT | 0.7790 | 0.9728 | 0.1487 |
| CRP | 0.3604 | 0.1474 | 0.1619 |
| EOS | 0.8489 | 0.9674 | 0.7545 |
| EOSLE | 0.7872 | 0.6208 | 0.9671 |
| HGB | 0.9671 | 0.7830 | 0.2552 |
| K | 0.6269 | 0.6798 | 0.9358 |
| LYM | 0.3341 | 0.8489 | 0.5626 |
| LYMLE | 0.4656 | 0.4282 | 0.9671 |
| MONO | 0.8753 | 0.6208 | 0.4871 |
| MONOLE | 0.4391 | 0.1117 | 0.9358 |
| NEUT | 0.0894 | 0.0894 | 0.4771 |
| NEUTLE | 0.2226 | 0.0894 | 0.6441 |
| PLAT | 0.9674 | 0.7872 | 0.2133 |
| RETI | 0.2552 | 0.6208 | 0.6208 |
| SODIUM | 0.7872 | 0.7830 | 0.9500 |
| WBC | 0.0894 | 0.1487 | 0.4494 |

**Supplementary Table 6: P values from paired tests comparing neutrophil-to-lymphocyte ratio (NLR) at baseline and six months within each cluster and treatment arm.**

| **Cluster** | **Treatment Arm** | **P value** |
| --- | --- | --- |
| 1 | Placebo | 0.955 |
| 1 | Treatment | <0.001 |
| 2 | Placebo | 0.325 |
| 2 | Treatment | 0.003 |
| 3 | Placebo | 0.148 |
| 3 | Treatment | 0.126 |


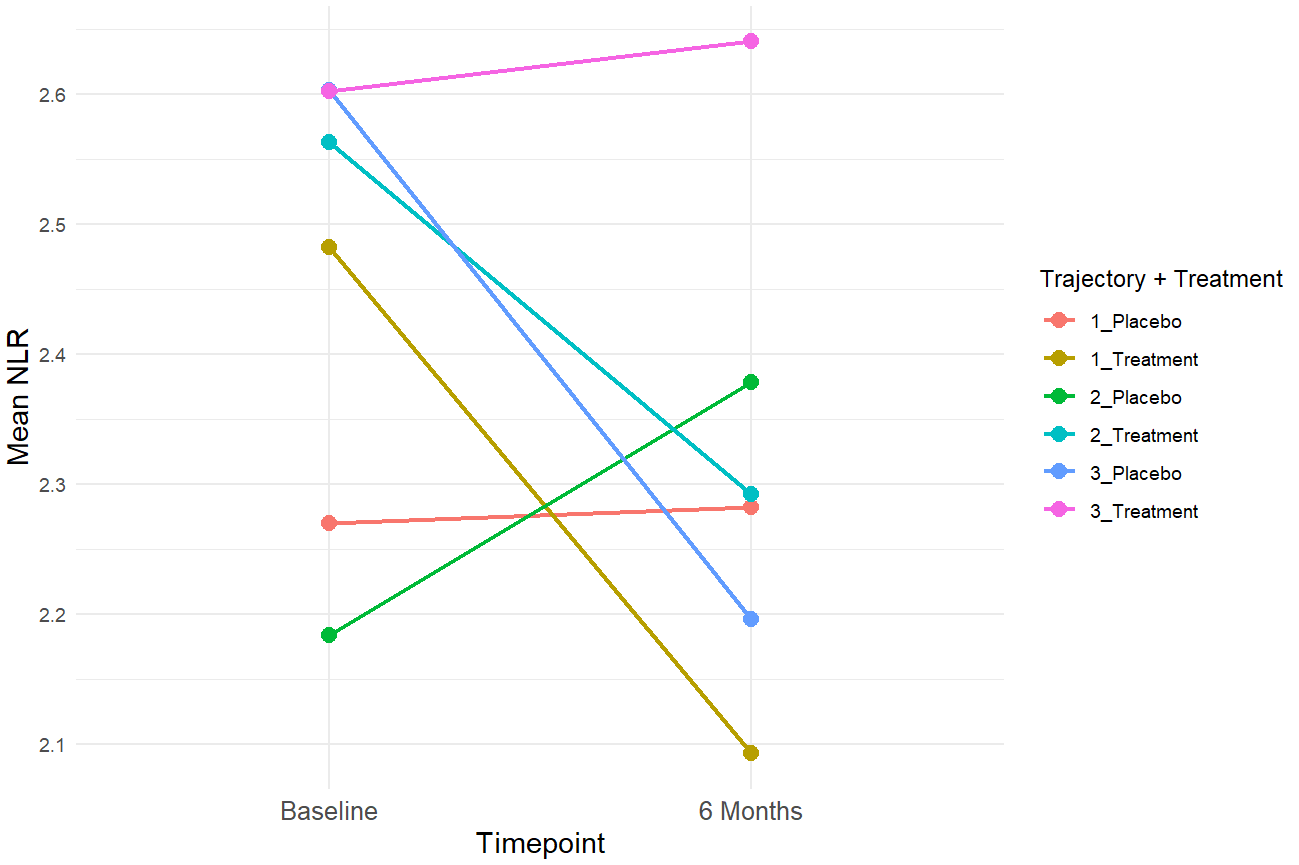


**Supplementary Figure 1: Mean Neutrophil-to-Lymphocyte Ratio (NLR) at Baseline and 6 Months by Trajectory Cluster and Treatment Group.** Line plot displays changes in mean NLR over time across trajectory clusters (1, 2, 3), stratified by treatment (solid lines) and placebo (dashed lines) groups.
